# Pre-analytic factors and initial biomarker levels in community-acquired pneumonia patients

**DOI:** 10.1186/1471-2253-14-102

**Published:** 2014-11-15

**Authors:** Alexander Kutz, Eva Grolimund, Mirjam Christ-Crain, Robert Thomann, Claudine Falconnier, Claus Hoess, Christoph Henzen, Werner Zimmerli, Beat Mueller, Philipp Schuetz, for the ProHOSP Study Group

**Affiliations:** University Department of Medicine, Tellstrasse, CH-5001 Kantonsspital Aarau, Switzerland; Department of Internal Medicine, Division of Endocrinology, Diabetes and Clinical Nutrition, University Hospital Basel, Basel, Switzerland; Department of Internal Medicine, Bürgerspital Solothurn, Solothurn, Switzerland; Basel University Medical Clinic Liestal, Liestal, Switzerland; Department of Internal Medicine, Kantonsspital Münsterlingen, Münsterlingen, Switzerland; Department of Internal Medicine, Kantonsspital Lucerne, Lucerne, Switzerland

**Keywords:** Community-acquired pneumonia, Blood biomarkers, Procalcitonin, C-reactive protein, White blood cells count, Proadrenomedullin, Copeptin, Pre-analytic factors, Pretreatment

## Abstract

**Background:**

Blood biomarkers are increasingly used to diagnose, guide therapy in, and risk-stratify community-acquired pneumonia (CAP) patients in emergency departments (EDs). How pre-analytic factors affect these markers’ initial levels in this population is unknown.

**Methods:**

In this secondary analysis of consecutive ED patients with CAP from a large multicentre antibiotic stewardship trial, we used adjusted multivariate regression models to determine the magnitude and statistical significance of differences in mean baseline concentrations of five biomarkers (procalcitonin [PCT], C-reactive protein [CRP], white blood cells count [WBC], proadrenomedullin [ProADM], copeptin) associated with six pre-analytic factors (antibiotic or corticosteroid pretreatment, age, gender, chronic renal failure or chronic liver insufficiency).

**Results:**

Of 925 CAP patients (median age 73 years, 58.8% male), 25.5% had antibiotic pretreatment, 2.4%, corticosteroid pretreatment, 22.3%, chronic renal failure, 2.4% chronic liver insufficiency. Differences associated with pre-analytic factors averaged 6.1% ±4.6%; the three largest statistically significant changes (95% confidence interval) were: PCT, +14.2% (+2.1% to +26.4%, p = 0.02) with liver insufficiency; ProADM, +13.2% (+10.2% to +16.1%, p < 0.01) with age above median; CRP, -12.8% (-25.4% to -0.2%, p = 0.05) with steroid pretreatment. In post hoc sensitivity analyses, reclassification statistics showed that these factors did not result in significant changes of biomarker levels across clinically used cut-off ranges.

**Conclusions:**

Despite statistically significant associations of some pre-analytic factors and biomarker levels, a clinically relevant influence seems unlikely. Our observations reinforce the concept of using biomarkers in algorithms with widely-separated cut-offs and overruling criteria considering the entire clinical picture.

**Trial registration:**

Identifier ISRCTN95122877.

**Electronic supplementary material:**

The online version of this article (doi:10.1186/1471-2253-14-102) contains supplementary material, which is available to authorized users.

## Background

To improve outcomes in community-acquired pneumonia (CAP), management guidelines emphasize early diagnosis to enable a timely start of appropriate antimicrobial therapy [1, 2]. For this purpose, circulating levels of biomarkers associated with bacterial infection and inflammation, procalcitonin (PCT), as well as C-reactive protein (CRP) and white blood cells count (WBC), are increasingly considered in the initial assessment of patients with signs or symptoms suggestive of CAP [3–5]. Additionally, biomarkers mirroring disease-related stress, physiological reserve, or both, are more often being used to stratify disease severity and risk of patients with CAP, to aid in triage and in site-of-care decision-making [6–9]. These analytes include proadrenomedullin (ProADM) or copeptin, which are stoichiometrically co-secreted with adrenomedullin or vasopressin, respectively.

Before admission, CAP patients presenting to the emergency department (ED) frequently have received pretreatment with antibiotics or corticosteroids. Further, these patients are often elderly and may have varying degrees of kidney or liver dysfunction. Such pre-analytic factors conceivably could affect initial biomarker levels and hence confound assay interpretation or require adjustment in analysing cut-offs. For example, hepatocyte malfunction affects the main site of CRP synthesis, renal failure could lead to accumulation of proteins normally excreted in the urine, and elderly patients might have a suppressed inflammatory response [10].

The presence and magnitude of any differences in concentrations of newer biomarkers that may be associated with pre-analytic factors in patients with CAP is important [11–17]. Previous literature has addressed such associations only in healthy volunteers or critical care patients, populations differing greatly from ED patients with CAP in pretreatment, age, gender composition, comorbidity, and general state [12–14, 18].

We therefore sought to characterise in a large, representative, and well-defined CAP cohort the relative differences in initial levels of five widely used diagnostic or prognostic blood biomarkers that were associated with each of six common and potentially important pre-analytic factors. We hypothesised that the magnitude and statistical significance of any changes would reflect the clinical relevance of the relationships and the possible need to consider adjusted cut-offs in subgroups in everyday practice. The five blood biomarkers investigated were PCT, CRP, WBC, ProADM, and copeptin, and the six pre-analytic factors were antibiotic or steroid pretreatment, age, gender, chronic renal failure, and chronic liver insufficiency.

## Methods

### Patients, setting, ethics

This secondary analysis included all patients with confirmed CAP from the completed prospective, multicentre, randomised, controlled ProHOSP study [19]. ProHOSP assessed PCT-guided antibiotic stewardship in consecutive adults (age ≥18 years) with presumed lower respiratory infection of <28 days’ duration presenting to EDs at any of six Swiss secondary or tertiary care, academic or non-academic hospitals from October 2006 to March 2008 [20].

Patients had to have come from the community or a nursing home with at least one symptom of cough, sputum production, dyspnoea, tachypnoea, or pleuritic pain, plus rales during auscultation or at least one infectious sign (core body temperature >38.0°C, shivering, WBC >10 or <4 cells × 10^9^/L). CAP was confirmed in all patients by new or increasing radiographic lung infiltrate. Patients were excluded for active intravenous drug abuse, severe immunosuppression other than corticosteroids, imminently life-threatening comorbidity, hospital-acquired pneumonia, or ongoing chronic antibiotic administration. Short-term antibiotic therapy or corticosteroid treatment before presentation did not affect eligibility. Within the trial, patients were stratified by study center and randomised 1:1 to antibiotic administration according to either i) state-of-the-art evidence-based guidelines (controls) or ii) an algorithm recommending antibiotics only when PCT values exceeded previously validated cut-offs [20].

Data on demographics, comorbidities, laboratory and vital parameters, imaging, current and recent medication, and other baseline characteristics were assembled for each patient upon presentation. Antibiotic or steroid pretreatment and comorbidities were identified through one or more of patient self-report, general practitioner notes and medical chart review. We did not collect data on diagnostic procedures such as CT scans performed during the trial, as such exams took place at the discretion of the treating physicians rather than systematically.

Approval for ProHOSP was obtained from all local ethical committees; patients gave written informed consent. ProHOSP was registered in the "Current Controlled Trials Database" ( http://www.controlled-trials.com/ISRCTN95122877).

### Biomarker measurement

In all patients, blood specimens for later marker measurement were collected upon admission, i.e., within the first 24 hours post-presentation. CRP concentrations were determined by an enzyme immunoassay (EMIT, Merck Diagnostica, Zürich, Switzerland) having a <5 mg/L detection limit. PCT determinations were made using a time-resolved amplified cryptate emission technology-based assay (Kryptor® PCT, Thermo Scientific Biomarkers [B R A H M S AG], Hennigsdorf, Germany) with a 0.06 μg/L functional sensitivity [21]. ProADM and copeptin were batch-measured in plasma with sandwich immunoassays (Kryptor®, Thermo Scientific Biomarkers) with 0.08 nmol/L and 0.4 pmol/L analytical detection limits, respectively [15–17, 22].

### Statistics

Our primary analysis used linear regression models to investigate the association of each studied pre-analytic factor with the level of each studied biomarker at ED presentation. Due to non-linearity, biomarker values were transformed into deciles before entry into the models. Regression coefficients thus correspond to a decile increase or decrease.

To investigate the potential influence of antibiotic pretreatment on biomarker concentrations, we compared the patients who at ED presentation, had any recent antibiotic exposure, regardless of agent(s), dose(s) or administration route(s), versus the patients without such exposure. For our analysis of the potential impact of corticosteroid pretreatment on biomarker levels, we compared patients receiving at least one dose of ≥20 mg prednisolone equivalent/day before presentation versus patients without such treatment. This threshold was chosen considering dose levels found to be relevant in prior studies focusing on corticosteroid influence on biomarker release [14, 23]. To analyse the influence of age, we formed two groups by dichotomising patients according to the median age of the study sample, 73 years. Since the ProHOSP protocol did not call for routine comprehensive evaluation of kidney or liver function at presentation, patients were dichotomised as to whether or not their medical records noted chronic renal failure or chronic liver insufficiency.

Regression models were adjusted for the following covariates: pneumonia severity index (PSI) [24], creatinine (transformed into deciles due to non-linearity), presence of chronic obstructive pulmonary disease (COPD), and presence of diabetes mellitus. The PSI is a well-validated CAP severity/risk scoring system based on a total of 20 sociodemographic variables, comorbidities (neoplasia, congestive heart failure, and hepatic, cerebrovascular or renal disease), and physical, radiographic, and laboratory findings (not including any of our studied biomarkers). Incorporating PSI as a covariate therefore adjusted for the patient’s CAP severity and simultaneously, comorbidity and general state. However, to prevent over-adjustment, for the analyses regarding the associations of the respective factors and biomarker levels, we used PSI scores excluding points for age, gender, or renal or hepatic dysfunction-related variables.

We also conducted exploratory analyses examining the influence of each of the six factors of interest on each of the five biomarker concentrations during a 1-week follow-up (day 0 [presentation], 3, 5, and 7 values); methodology is described elsewhere (see Additional file 1). To evaluate the clinical impact of biomarker level changes, in post hoc sensitivity analyses, reclassification statistics were performed (see Additional file 2).

All confidence intervals (CIs) are two-sided; tests were carried out at 5% significance levels. Analyses were performed with STATA 12.1 (Stata Corp., College Station, TX, USA). Discrete variables are expressed as counts, percentages, or both, and continuous variables are expressed as medians and interquartile ranges (IQRs; 25^th^-75^th^ percentiles).

## Results

The study sample comprised all 925 confirmed CAP patients included in the ProHOSP trial. Baseline characteristics are summarised in Table 1. Antibiotic pretreatment was common, administered to just over one-quarter of the study sample, whilst steroid pretreatment was uncommon (2.4% of patients). Typical of the ED population with CAP, the study sample tended to be elderly and male and had a substantial comorbidity burden, which included chronic renal failure in a bit over one-fifth of cases, but chronic liver failure in relatively few patients (2.4%).

**Table 1:** **Baseline characteristics of the study sample**

Characteristics	All patients (N = 925)
***Demographic characteristics***	
Age, median (IQR), yr.	73 (59-82)
Male, % (n)	58.8% (544)
***Coexisting illnesses***, % **(** ***n*** **)** ^a^	
Chronic renal failure	22.3% (206)
Chronic liver insufficiency	2.4% (22)
Chronic obstructive pulmonary disease	30.5% (282)
Diabetes mellitus	17.5% (162)
Congestive heart failure	17.2% (159)
***Clinical history***, % (***n***)	
Antibiotic pretreatment^a,b^	25.5% (236)
Steroids **≥**20 mg prednisolone equivalent/d^a^	2.4% (22)
Cough	82.3% (761)
Sputum	47.2% (437)
Fever	66.8% (618)
Chill	32.5% (301)
Dyspnoea	75.1% (695)
New York Heart Association class at presentation	
I	9.8% (91)
II	27.2% (252)
III	26.1% (241)
IV	12.0% (111)
***Clinical findings***	
Confusion, % (n)	8.0% (74)
Rales during auscultation, % (n)	68.8% (636)
Pneumonia severity index, median (IQR)^c^	92 (68-116)
Respiratory rate, median (IQR), breaths/min	20 (16-25)
Systolic blood pressure, median (IQR), mmHg	132 (119-148)
Diastolic blood pressure, median (IQR), mmHg	74 (65-83)
Heart rate, median (IQR), beats/min	95 (82-108)
Body temperature, median (IQR), °C	38.1 (37.2-38.9)
***Initial laboratory findings***, ***median*** ***(IQR)***	
PCT, μg/L	0.46 (0.15-2.66)
C-reactive protein, mg/dL	155 (75-252)
White blood cells count, cells × 10^9^/L	12.1 (9-16.4)
ProADM, nmol/L	1.2 (0.81-1.86)
Copeptin, pmol/L	25 (12.7-51.1)
Creatinine, μmol/L	89 (69-113)
***Treatment site***, % **(** ***n*** **)**	
Inpatient	91.2% (844)

IQR, interquartile range; PCT, procalcitonin; ProADM, proadrenomedullin.

Due to inclusion criteria for the present analysis, all patients had a history of CAP.

^a^All data on comorbidities and pretreatments were based on patient report and medical chart review.

^b^Includes patients receiving at least one dose of antibiotics irrespective of agent, regimen or administration route.

^c^The pneumonia severity index [24] is scored based on 20 sociodemographic, clinical, laboratory, or comorbidity variables. Patients are categorised into 5 classes, with increasing scores and classes denoting worse severity and mortality risk.

**Figure 1:**
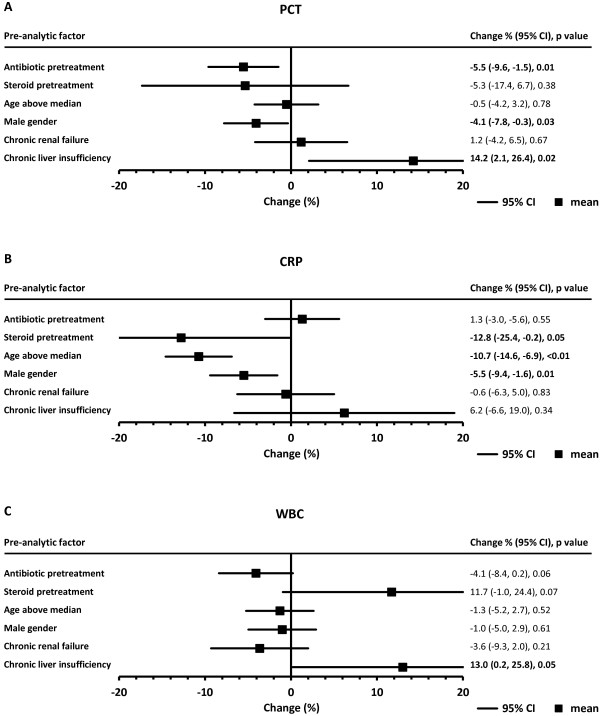
**Forest plots of mean relative differences (%) in initial inflammatory biomarker levels at presentation depending on pretreatment, demographics, or comorbidity: (A) PCT, (B) CRP, (C) WBC.** PCT, procalcitonin; CI, confidence interval; CRP, C-reactive protein; WBC, white blood cells count; error bars are 95% CIs. Values of the differences are given in the right-hand column, with significant differences in bold text.

**Figure 2:**
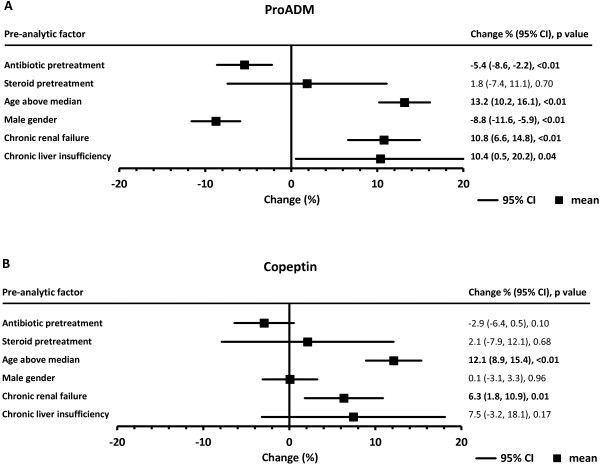
**Forest plots of mean relative differences (%) in initial stress/physiologic reserve biomarker levels at presentation depending on pretreatment, demographics, or comorbidity: (A) ProADM, (B) copeptin.** CI, confidence interval; ProADM, proadrenomedullin; error bars are 95% CIs. Values of the differences are given in the right-hand column, with significant differences in bold text.

Figures 1 and 2 represent graphically, whilst Table 2 lists, adjusted relative differences in initial biomarker concentrations between subgroups with or without the studied pre-analytic factors, and the 95% CI of those differences.

**Table 2:** **Adjusted percentage relative change** (**95**% **CI**), **initial biomarker concentrations for selected baseline characteristics relative to absence of the characteristic**

Biomarker	Antibiotic pretreatment	Steroid pretreatment	Age above study sample median	Male gender	Chronic renal failure	Chronic liver insufficiency ^a^
	(n = 236) ^a,b^	(n = 22) ^a,c^	(n = 462)	(n = 544)	(n = 206) ^a^	(n = 22) ^a^
**PCT**	-**5.5**% (-**9.6**%, -**1.5**%), **p** = **0.01**	-5.3% (-17.4%, 6.7%), p = 0.38	-0.5% (-4.2%, 3.2%), p = 0.78	-**4.1**% (-**7.8**%, -**0.3**%), **p** = **0.03**	1.2% (-4.2%, 6.5%), p = 0.67	**14.2**% (**2.1**%, **26.4**%), **p** = **0.02**
**CRP**	1.3% (-3.0%, 5.6%), p = 0.55	-**12.8**% (-**25.4**%, -**0.2**%), **p** = **0.05**	-**10.7**% (-**14.6**%, -**6.9**%), **p** < **0.01**	-**5.5**% (-**9.4**%, -**1.6**%), **p** = **0.01**	-0.6% (-6.3%, 5.0%), p = 0.83	6.2% (-6.6%, 19.0%), p = 0.34
**WBC**	-4.1% (-8.4%, 0.2%), p = 0.06	11.7% (-1.0%, 24.4%), p = 0.07	-1.3% (-5.2%, 2.7%), p = 0.52	-1.0% (-5.0%, 2.9%), p = 0.61	-3.6% (-9.3%, 2.0%), p = 0.21	**13.0**% (**0.2**%, **25.8**%), **p** = **0.05**
**ProADM**	-**5.4**% (-**8.6**%, -**2.2**%), **p** < **0.01**	1.8% (-7.4%, 11.1%), p = 0.70	**13.2**% (**10.2**%, **16.1**%), **p** < **0.01**	-**8.8**% (-**11.6**%, -**5.9**%), **p** < **0.01**	**10.8**% (**6.6**%, **14.8**%), **p** < **0.01**	**10.4**% (**0.5**%, **20.2**%), **p** = **0.04**)
**Copeptin**	-2.9% (-6.4%, -0.5%), p = 0.10	2.1% (-7.9%,12.1%), p = 0.68	**12.1**% (**8.9**%, **15.4**%), **p** < **0.01**	-0.1% (-3.1%, 3.3%), p = 0.96	**6.3**% (**1.8**%, **10.9**%), **p** = **0.01**	7.5% (-3.2%, 18.1%), p = 0.17)

CI, confidence interval; CRP, C-reactive protein; PCT, procalcitonin; WBC, white blood cells count; ProADM, proadrenomedullin.

Values for baseline factors associated with a statistically significant adjusted relative change in the initial biomarker level are in bold type.

^a^All data on comorbidities and pretreatments were based on patient report and medical chart review.

^b^Includes patients receiving at least one dose of antibiotics irrespective of agent, regimen, or administration route.

^c^≥ 20 mg/d prednisolone equivalent.

### PCT

Adjusted relative differences in mean baseline PCT between those with the studied pre-analytic factors versus those without those factors ranged from -5.5% (antibiotic pretreatment) to 14.2% (chronic liver insufficiency). Older age showed the smallest impact on PCT levels (-0.5%). Three factors were associated with a significantly different initial mean PCT concentration: antibiotic pretreatment or being male with lower values, and chronic liver insufficiency with a higher value (Figure 1A).

### CRP

For average initial CRP, adjusted relative differences between patients with the studied pre-analytic factors versus their counterparts without these factors ranged from -12.8% (steroid pretreatment) to 6.2% (chronic liver insufficiency). Almost no difference was seen in patients with chronic renal failure (-0.6%). The differences in mean baseline CRP were statistically significant for three factors: pretreatment with steroids, older age, or male gender-all were associated with lower levels (Figure 1B).

### WBC

Adjusted differences in initial WBC associated with the studied pre-analytical factors varied between -4.1% (antibiotic pretreatment) to 13.0% (chronic liver insufficiency). The smallest difference observed was for male gender (-1.0%). Chronic liver failure was associated with significantly elevated mean initial leukocyte count; WBC tended to be lower in patients receiving prior antibiotics and higher in those given prior corticosteroids than in those not administered the respective pretreatment (Figure 1C).

### ProADM

The minimum difference in initial ProADM associated with the studied pre-analytic factors was 1.8% (steroid pretreatment) and the maximum was 13.2% (older age). Differences were significant for five of six studied factors (Figure 2A): antibiotic pretreatment or male gender were associated with lower mean ProADM at presentation, whilst increasing age, chronic renal failure, or chronic liver insufficiency were associated with higher ProADM concentrations at that time.

### Copeptin

Changes in baseline copeptin levels associated with the studied factors ranged from -2.9% (antibiotic pretreatment) to 12.1% (older age). Male gender showed the smallest effect (-0.1%). The changes attained statistical significance for two factors, increasing age and chronic renal failure, both of which were associated with copeptin elevations (Figure 2B).

## Discussion

The most important and reassuring finding of this secondary analysis was that, despite statistical significance of some associations, none of the six studied pre-analytic factors individually seemed to be linked to dramatic relative changes in mean initial levels of any of the five studied biomarkers. The relative differences in baseline biomarker concentration between patients with versus without the factors were statistically significant in 14/30 cases. However, irrespective of whether patients with the pre-analytic factors had elevated or decreased biomarker levels, the 30 differences themselves ranged from 0.1% to 14.2% and averaged 6.1% ±4.6% (9.5% ±3.5% for the 14 significant differences), and even their 95% CIs never included a >27% change.

These observations support the current concept of using blood biomarkers in validated algorithms with a few widely separated cut-offs, without formal adjustment for pre-analytic factors [4, 6]. At least regarding antibiotic stewardship in CAP, this concept has proved clearly successful. For example, PCT algorithms developed in clinical trials to recommend or strongly recommend for/against starting antibiotics in patients presenting to the ED with presumed CAP generally applied PCT cut-offs of 0.1 μg/L, 0.25 μg/L, and 0.5 μg/L [6]; a proposed algorithm for CAP severity/risk classification using ProADM plus a clinical scoring system contains ProADM cut-offs of 0.75 nmol/L and 1.5 nmol/L [8, 25]. Where higher-risk or higher-severity clinical categories are delineated by a doubling or more in biomarker concentrations, changes of 4.1% or even 26.4% (the range in magnitude of significant differences and their 95% CIs) that may be associated with an individual pre-analytic factor would seem unlikely to produce clinically relevant confounding. This assumption was further evidenced in post-hoc sensitivity analyses, namely reclassification analyses, using different biomarker specific cut-off ranges and estimates from the above-mentioned models (see Additional file 2).

At the same time, our present observations support the concept that biomarker values should not be used in isolation. Rather, these values should be considered in conjunction with the patient’s clinical presentation and history, imaging and other laboratory results, etc., as well as in light of the physician’s clinical experience and judgment. One way that present biomarker algorithms acknowledge this concept is by including "overruling criteria", i.e., provision to override on a variety of clinical, patient psychosocial, or logistical grounds recommendations based strictly, or largely, on analytic levels [6, 8]. The overruling criteria could provide a "safety net" in patients presenting with a number of pre-analytic factors that in aggregate, if not individually, might materially affect biomarker levels.

Our results are generally in line with previous observations. For example, Krüger et al. noted lower levels of PCT and WBC but not of CRP in patients with antibiotic pretreatment [11]. Schaaf et al. found slightly decreased admission CRP levels in patients with prior antimicrobial therapy [26]. In our analysis, this association only became significant when considering CRP values during follow-up (see Additional files 1 and 3), perhaps due at least partly to the delayed kinetics of CRP versus PCT. Importantly, the Schaaf study was not adjusted for confounders other than antibiotic pretreatment, which also may help to explain differences with our results. Similar to our observations, two intensive care unit studies found decreased CRP levels after methylprednisolone treatment, reflecting the suppressive effect of corticosteroids on inflammatory markers [12, 13]. Remarkably, no such effects on PCT were observed in our study or other studies [12, 14]. Additionally, Bruns and colleagues found an influence of inadequate antibiotic treatment on CRP levels [27]. Because in our cohort, we only had an 8% rate of positive blood cultures, with very few multi-resistant bacteria [28], we were unable to examine this factor.

In older patients, our analysis revealed significantly lower mean initial CRP levels. This finding might be explained by the inverse correlation of inflammatory response and age [10, 29].

Renal clearance is one of the PCT elimination pathways [30]. However, the lack of significant change in our investigated inflammatory biomarker levels in patients with chronic renal failure also agrees with earlier results [30–33]. Those data showed that urinary PCT levels were significantly reduced in patients with severe renal dysfunction. However, despite decreased renal elimination, the plasma PCT clearance rate correlated only weakly with renal dysfunction, and thus interpretation of plasma PCT levels should not be confounded by this condition.

In patients with chronic liver insufficiency, we found significantly elevated PCT levels. Similarly, Elefsiniotis et al. noted that significant proportions of patients with acute alcoholic hepatitis on a cirrhotic background or with acute on chronic viral hepatitis but without bacterial infection, exhibited serum PCT levels above 0.5 μg/L [34].

We are aware of several limitations of the present work. Firstly, this was a secondary analysis of a previous antibiotic stewardship trial. Therefore, clinical information about pretreatments (dose and duration) and comorbidities were not always systematically collected. Consequently, our analysis dichotomised patients broadly with respect to prior antibiotics or corticosteroids, without considering the type or regimen of these pretreatments. Nevertheless, although data on indications for these modalities were not formally collected, our clinical impression was that antibiotic pretreatment seemed almost always to have represented attempts by general practitioners to address the respiratory infections eventually precipitating the ED visits. Additionally, steroid pretreatment seemed to have been given primarily as therapy for pre-existing COPD. Another disadvantage of the secondary analysis was that chronic renal failure and chronic liver insufficiency were not classified by severity, duration, or aetiology. All these factors may have led to patient misclassification. Additionally, given these limitations to a "pristine" analysis, we did not correct for multiple comparisons or adjust for potential interactions between or among studied pre-analytic factors. Rather, we sought to detect material changes in baseline concentrations of widely used biomarkers associated with key pre-analytic factors whilst adjusting for important potential confounders, namely pneumonia severity and comorbidity. Therefore, synergistic or compensating effects of multiple pre-analytic factors in a given patient cannot be completely excluded based on our results.

Secondly, numbers of patients with prior corticosteroid treatment or chronic liver insufficiency were small (n = 22/925 each), leading to potential under-powering of related analyses. Nonetheless, we consistently observed a stable trend of biomarker values in these subgroups during the 1-week follow-up, a finding that appears to render random fluctuation rather unlikely (see Additional files 1, 3 and 4).

## Conclusion

Each studied pre-analytic factor appeared to be associated with a statistically significant relative change in mean baseline levels of one or more of the examined biomarkers. However, such changes averaged only 6.1% overall and 9.5% when statistically significant, and never exceeded 15%, or, with respect to the outer bounds of the 95% CIs, 27%. Our findings therefore provide reassurance that adjustment for antibiotic or corticosteroid pretreatment, older age, male gender, or chronic renal failure or liver insufficiency are in most cases unlikely to be necessary when biomarkers are used in algorithms containing a few widely separated cut-offs as well as overruling criteria allowing consideration of the entire clinical picture.

## Electronic supplementary material

Additional file 1: **Pre-analytic factors and initial biomarker levels in community-acquired pneumonia patients – supplementary material.** (DOC 36 KB)

Additional file 2: **Reclassification of inflammatory biomarkers with significant adjusted relative changes for evaluation of clinical impact.** (DOC 34 KB)

Additional file 3: **Mean relative changes (%) in baseline, day 3, day 5, and day 7 levels of PCT (A), CRP (B) and WBC (C) associated with antibiotic and corticosteroid pretreatment, age, gender, chronic renal failure and chronic liver insufficiency.** (PDF 174 KB)

Additional file 4: **Mean relative changes (%) in baseline, day 3, day 5, and day 7 levels of ProADM (A) and copeptin (B) associated with antibiotic and corticosteroid pretreatment, age, gender, chronic renal failure and chronic liver insufficiency.** (PDF 171 KB)

## Abbreviations

CAP: Community-acquired pneumonia
CI: Confidence interval
COPD: Chronic obstructive pulmonary disease
CRP: C-reactive protein
ED: Emergency department
IQR: Interquartile range
PCT: Procalcitonin
ProADM: Proadrenomedullin
PSI: Pneumonia severity index
WBC: White blood cells count.
