# Supplementary material for: Pre-analytic factors and initial biomarker levels in community-acquired pneumonia patients
Source: BMC Anesthesiol. 2014 Nov 15;14:102. doi: 10.1186/1471-2253-14-102 (PMC4240803; doi:10.1186/1471-2253-14-102)
Supplement: Supplementary file 1 — Additional file 1: Pre-analytic factors and initial biomarker levels in community-acquired pneumonia patients – supplementary material. (DOC 36 KB) [file 12871_2014_316_MOESM1_ESM.doc]

**Pre-analytic factors and initial biomarker levels in**

**community-acquired pneumonia patients – supplementary material**

**Methods**

In an exploratory analysis, we also examined the association of the six studied pre-analytic factors and differences in day 3, day 5, and day 7 concentrations of the five studied blood biomarkers. The methodology was the same as described for the primary analysis in the Methods section of the main manuscript, except that no adjustments were made for pneumonia severity, comorbidities, or general status. To deal with missing values for biomarkers or the PSI, we used multiple imputation by chained equations, with the imputation dataset comprising all CAP patients.

**Results**

**Supplementary Figure 1** shows the results of the exploratory analysis for PCT (Panel A), CRP (Panel B) and WBC (Panel C), **Supplementary Figure 2,** those for ProADM (Panel A) and copeptin (Panel B).

*PCT (****Supplementary Figure 1A****)*

The statistically significant association of antibiotic pretreatment with reduced PCT levels seen for the initial measurement continued for the three follow-up measurements. Steroid pretreatment and age above the median continued to show the same lack of association with differences in biomarker levels for follow-up measurements as they had for the baseline determination. Male gender was no longer associated with any statistically significant change in biomarker levels post-presentation. By contrast, chronic renal failure was associated with significant elevation in day 7 PCT values. The relationship of chronic liver insufficiency and significantly higher PCT values seen at presentation also was present for day 5 measurements, but not day 3 or 7 values. Relative changes in PCT concentrations associated with follow-up measurements ranged from -9.1% (antibiotic pretreatment, day 3) to 12.4% (chronic liver insufficiency, day 5). The smallest difference during follow-up was observed in patients with chronic renal failure (1.0%, day 3).

*CRP (****Supplementary Figure 1B****)*

Though antibiotic pretreatment was not associated with significantly different CRP levels at baseline, this factor was associated with significantly lower CRP levels on days 3, 5, and 7. No other studied pre-analytic factor was associated with significantly different follow-up CRP levels, including steroid pretreatment, older age, and male gender, all of which were associated with significantly reduced baseline CRP levels. Relative changes in CRP concentrations associated with follow-up measurements ranged from -5.7% (antibiotic pretreatment, day 3) to 7.9% (chronic liver insufficiency, day 5).

*WBC (****Supplementary Figure 1C****)*

No pre-analytic factor displayed a relationship with altered follow-up WBC measurements, including chronic liver insufficiency, which had shown such an association at presentation. Relative differences in WBC concentrations associated with day 3-7 determinations ranged from -2.9% (older age, day 5) to 10.2% (chronic liver insufficiency, day 3).

*ProADM (****Supplementary Figure 2A****)*

For all follow-up measurements, older age and chronic renal failure continued the association with significantly elevated ProADM that was seen for the baseline concentration. Likewise, the association of male gender with significantly decreased ProADM that was seen at baseline was also observed on days 3, 5, and 7. Conversely, after presentation, neither antibiotic pretreatment nor chronic liver insufficiency was associated with any significant change in ProADM. The lack of such association with steroid pretreatment that was seen for initial values continued on days 3, 5, and 7. Relative changes in ProADM levels associated with day 3-7 determinations ranged from -8.5% (male gender, day 3) to 22.9% (older age, day 7).

*Copeptin (****Supplementary Figure 2B****)*

The associations of age above the median and chronic renal failure with significantly higher copeptin concentrations seen at baseline also were present for all three follow-up measurements. Whereas neither male gender nor chronic liver insufficiency was associated with significant differences in baseline copeptin, such a relationship was observed for male gender for day 5 and day 7 measurements and for liver dysfunction for day 7 values. Neither antibiotic nor steroid pretreatment was associated with any difference in copeptin follow-up measurements. Relative changes in copeptin values associated with day 3-7 determinations ranged from -3.7% (steroid pretreatment, day 5) to 14.9% (older age, day 3).

**Discussion**

The findings of this exploratory analysis should be interpreted particularly cautiously since they are subject not only to the present study’s limitations discussed in the main manuscript, but to three other potentially important limitations. Firstly, unlike in the main analysis, in this exploratory effort, no adjustment was made for the possible confounding factors of pneumonia severity, comorbidity, or general status. Secondly, no adjustment was made for medical treatment, particularly antibiotic or steroid therapy or supportive care for organ dysfunction, during the ProHOSP trial. This latter lack of adjustment may have introduced material bias in follow-up measurements, particularly with respect to the analyses involving antibiotics, steroids, and chronic renal failure or chronic liver insufficiency. Thirdly, to deal with missing values of biomarkers and PSI, multiple imputation was used. Although this is a well-accepted method, its use also may have introduced error.

These caveats should be kept in mind, but the findings of this exploratory analysis appear to support those of the main analysis. Although over the first week of care, some pre-analytic factors lost and others gained associations with significant differences in levels of the studied biomarkers, even the significant relative changes remained somewhat modest. Perhaps the most interesting finding of the exploratory analysis was that although CRP seemed to be unaffected by antibiotic pretreatment at baseline, presence of that factor became associated with significantly lower CRP on days 3, 5, and 7. The apparent delayed response to antibiotic pretreatment of CRP relative to that of PCT may reflect the kinetics of these biomarkers, underlining the earlier release of PCT compared to CRP.

**FIGURE LEGENDS**

**Supplementary Figure 1. Mean relative changes (%) in baseline, day 3, day 5, and day 7 levels of PCT (A), CRP (B) and WBC (C) associated with antibiotic and corticosteroid pretreatment, age, gender, chronic renal failure and chronic liver insufficiency.** *=p<0.05; **=p<0.01; ***=p<0.001; error bars represent standard error. CRP, C-reactive protein; PCT, procalcitonin; WBC, white blood cells count.

**Supplementary Figure 2. Mean relative changes (%) in baseline, day 3, day 5, and day 7 levels of ProADM (A) and copeptin (B) associated with antibiotic and corticosteroid pretreatment, age, gender, chronic renal failure and chronic liver insufficiency.** *=p<0.05; **=p<0.01; ***=p<0.001; error bars represent standard error. ProADM, proadrenomedullin.
