# Supplementary material for: Pre-analytic factors and initial biomarker levels in community-acquired pneumonia patients
Source: BMC Anesthesiol. 2014 Nov 15;14:102. doi: 10.1186/1471-2253-14-102 (PMC4240803; doi:10.1186/1471-2253-14-102)
Supplement: Supplementary file 2 — Additional file 2: Reclassification of inflammatory biomarkers with significant adjusted relative changes for evaluation of clinical impact. (DOC 34 KB) [file 12871_2014_316_MOESM2_ESM.doc]

**Supplementary Table 1. Reclassification of inflammatory biomarkers with significant adjusted relative changes for evaluation of clinical impact**

| **Biomarker** | **Antibiotic**  **pretreatment** **(n = 236)**^a,b^ | **Steroid pretreatment**  **(n = 22)**^a,c^ | **Age above study sample median (n = 462)** | **Male gender**  **(n = 544)** | **Chronic renal failure**  **(n = 206)**^a^ | **Chronic liver insufficiency**^a^  **(n = 22)**^a^ |
| --- | --- | --- | --- | --- | --- | --- |
| **PCT**  *Reclassified, % (n)*  *Not reclassified, % (n)* | 1.4 (13)  98.6 (912) | nd  nd | nd  nd | 1.7 (16)  98.3 (909) | nd  nd | 0.1 (1)  99.9 (924) |
| **CRP**  *Reclassified, % (n)*  *Not reclassified, % (n)* | nd  nd | 0.1 (1)  99.9 (924) | 3.2 (30)  96.8 (895) | 1.5 (14)  98.5 (911) | nd  nd | nd  nd |
| **WBC**  *Reclassified, % (n)*  *Not reclassified, % (n)* | nd  nd | nd  nd | nd  nd | nd  nd | nd  nd | 0 (0)  100 (925) |

PCT, procalcitonin; CRP, C-reactive protein; WBC, white blood cells count; nd, not done because no significant association was detected between the pre-analytic factor and the biomarker.

Reclassification of inflammatory biomarkers with initial statistically significant adjusted relative change: PCT, (PCT algorithm, cut-offs: 0.1 µg/L, 0.25 µg/L, 0.5 µg/L); CRP, (cut-offs: 50 mg/dL, 200 mg/dL); WBC (4 cells x 10^9^/L, 10 cells x 10^9^/L).

^a^All data on comorbidities and pretreatments were based on patient report and medical chart review.

^b^Includes patients receiving at least one dose of antibiotics irrespective of agent, regimen, or administration route.

^c^≥20 mg/d prednisolone equivalent.
