# Supplementary figures and images for: Pre-analytic factors and initial biomarker levels in community-acquired pneumonia patients
Source: BMC Anesthesiol. 2014 Nov 15;14:102. doi: 10.1186/1471-2253-14-102 (PMC4240803; doi:10.1186/1471-2253-14-102)

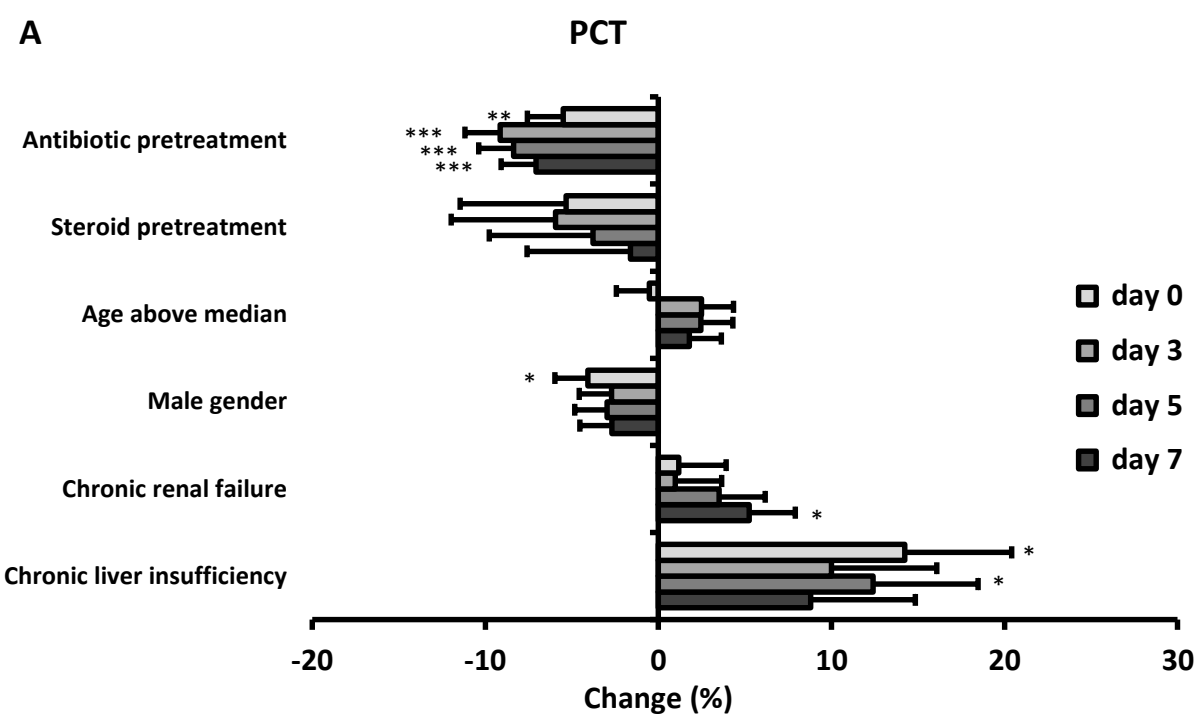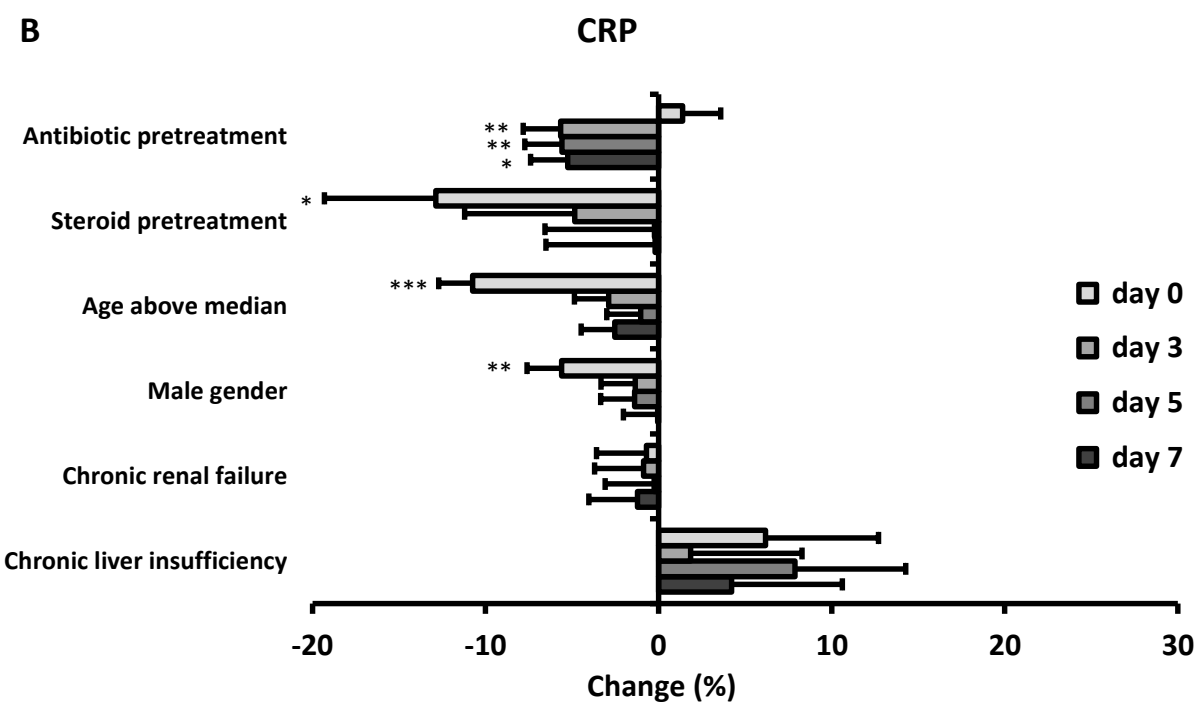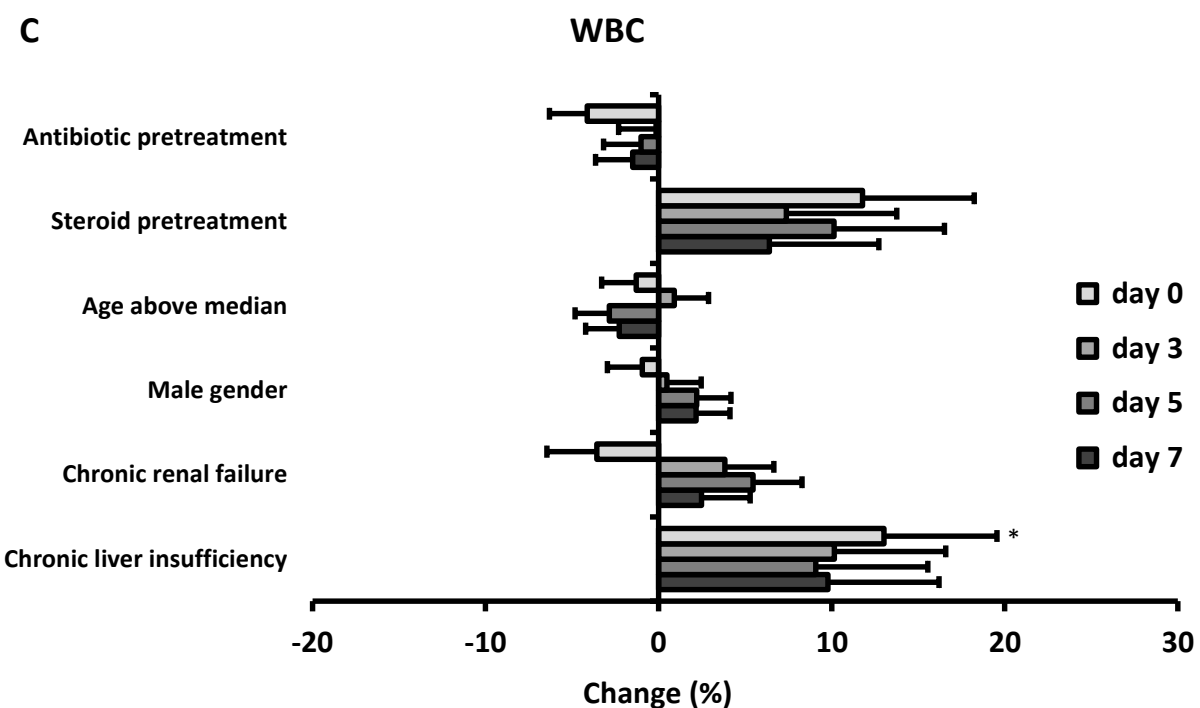

Supplement: Supplementary file 3 — Additional file 3: Mean relative changes (%) in baseline, day 3, day 5, and day 7 levels of PCT (A), CRP (B) and WBC (C) associated with antibiotic and corticosteroid pretreatment, age, gender, chronic renal failure and chronic liver insufficiency. (PDF 174 KB) [file 12871_2014_316_MOESM3_ESM.pdf]

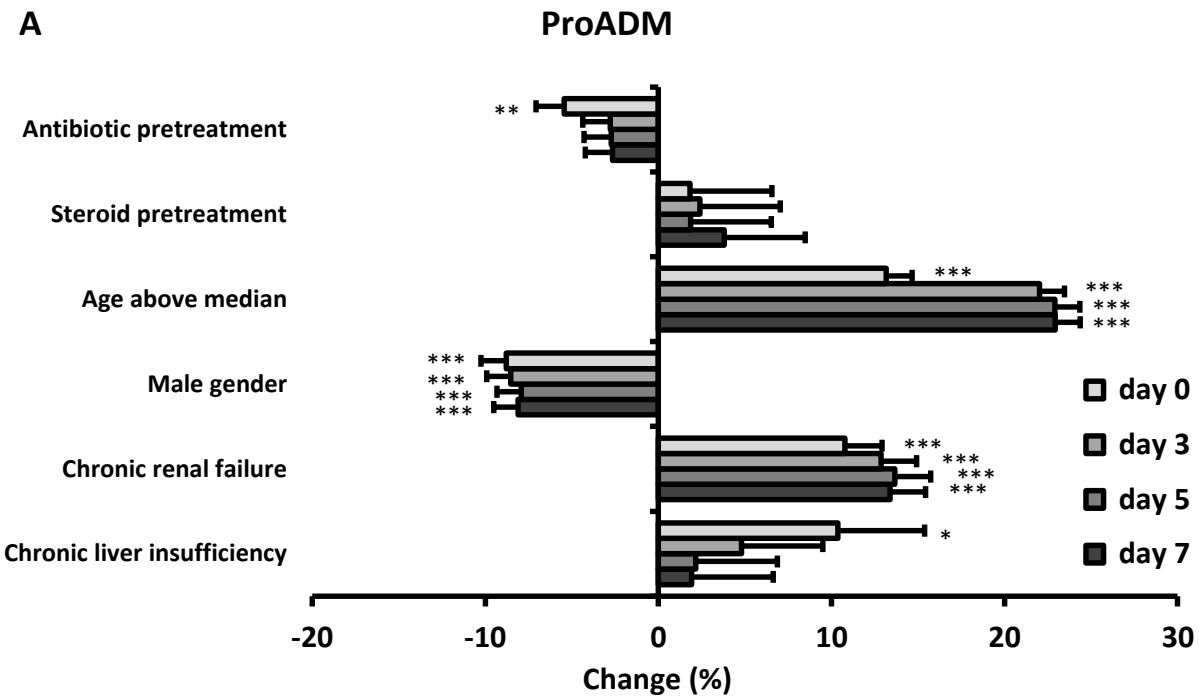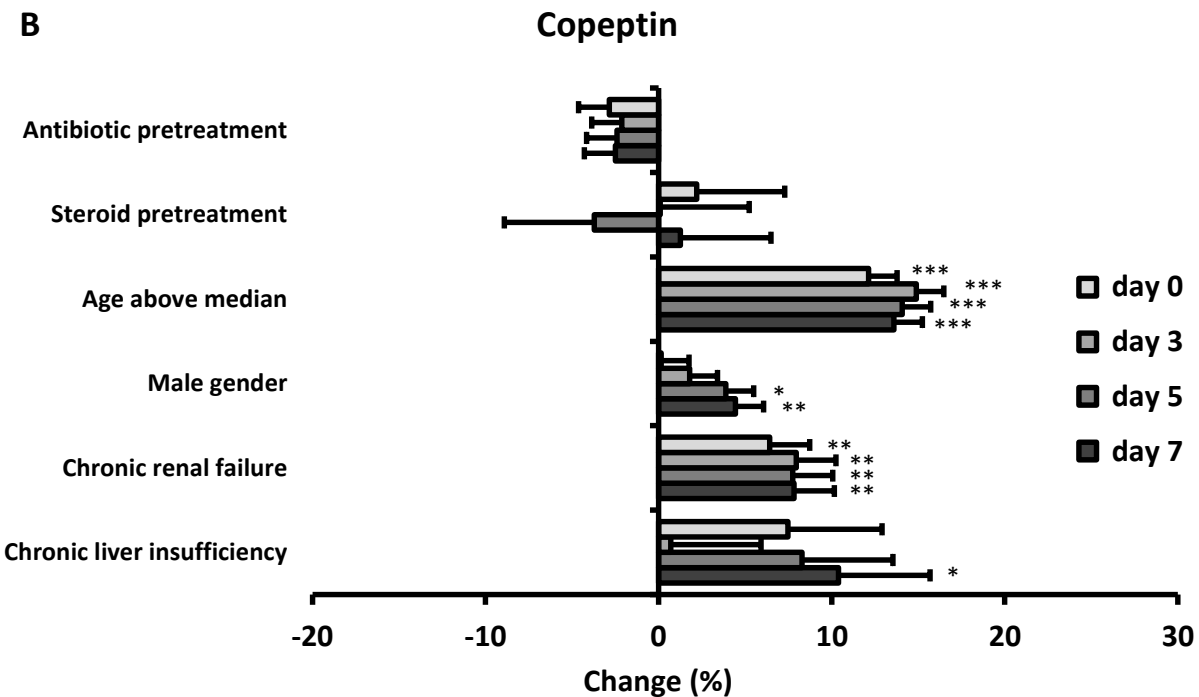

Supplement: Supplementary file 4 — Additional file 4: Mean relative changes (%) in baseline, day 3, day 5, and day 7 levels of ProADM (A) and copeptin (B) associated with antibiotic and corticosteroid pretreatment, age, gender, chronic renal failure and chronic liver insufficiency. (PDF 171 KB) [file 12871_2014_316_MOESM4_ESM.pdf]
